# Risk factors and in-hospital outcome of acute ST segment elevation myocardial infarction in young Bangladeshi adults

**DOI:** 10.1186/s12872-015-0069-2

**Published:** 2015-07-22

**Authors:** Mohammad Azizul Karim, Abdullah Al Shafi Majumder, Khandaker Qamrul Islam, Muhammad Badrul Alam, Makhan Lal Paul, Mohammad Shafiqul Islam, Kamrun N. Chowdhury, Sheikh Mohammed Shariful Islam

**Affiliations:** 1 National Institute of Cardiovascular Diseases (NICVD), Dhaka, Bangladesh; 2 Rangpur Medical College, Rangpur, Bangladesh; 3 Central Medical College, Comilla, Bangladesh; 4 Trishal Health Complex, Mymensingh, Bangladesh; 5 Department of Epidemiology, National Centre for Control of Rheumatic Fever and Heart Disease, Dhaka, Bangladesh; 6 International Center for Diarrhoeal Disease Research, Bangladesh, Center for Control of Chronic Diseases, Dhaka, Bangladesh; 7 Center for International Health, University of Munich, Munich, Germany; 8 Cardiovascular Division, The George Institute for Global Health, Sydney, Australia

## Abstract

**Background:**

South Asians have a higher overall incidence rate and younger age of onset for acute myocardial infarction (AMI) compared to Western populations. However, limited information is available on the association of preventable risk factors and outcomes of AMI among young individuals in Bangladesh. The aim of this study was to determine the risk factors and in-hospital outcome of AMI among young (age ≤40 years) adults in Bangladesh.

**Methods:**

We conducted a prospective observational study among consecutive 50 patients aged ≤40 years and 50 patients aged >40 years with acute ST Segment Elevation Myocardial Infarction (STEMI) and followed-up in-hospital at the National Institute of Cardiovascular Diseases (NICVD). Clinical characteristics, biochemical findings, diet, echocardiography and in-hospital outcomes were compared between the two groups. Multivariate logistic regression was performed to assess the association between risk factors and in-hospital outcome in young patients adjusting for other confounding variables.

**Results:**

The mean age of the young and older patient groups was 36.5 ± 4.6 years and 57.0 ± 9.1 years respectively. Male sex (OR 3.4, 95 % CI 1.2 − 9.75), smoking (OR 2.4, 95 % CI 1.04 − 5,62), family history of MI (OR 2.4, 95 % CI 1.11 − 5,54), homocysteine (OR 1.2, 95 % CI 1.08 − 1.36), eating rice ≥2 times daily (OR 3.5, 95 % CI 1.15 − 10.6) and eating beef (OR 4.5, 95 % CI 1.83 − 11.3) were significantly associated with the risk of AMI in the young group compared to older group. In multivariate analysis, older patients had significantly greater chance of developing heart failure (OR 7.5, 95 % CI 1.51 to 37.31), re-infarction (OR 7.0, 95 % CI 1.08 − 45.72), arrhythmia (OR 15.3, 95 % CI 2.69 − 87.77) and cardiogenic shock (OR 69.0, 95 % CI 5.81 − 85.52) than the younger group.

**Conclusion:**

Younger AMI patients have a different risk profile and better in-hospital outcomes compared to the older patients. Control of preventable risk factors such as smoking, unhealthy diet, obesity and dyslipidemia should be reinforced at an early age in Bangladesh.

## Background

Cardiovascular disease (CVD) is a global health problem reaching epidemic proportions in both developed and developing countries and it is the leading cause of mortality and morbidity worldwide [1, 2]. The South Asian countries have among the highest incidences of CVD globally [3]. Estimates from the global burden of disease study suggests that by the year 2020 this part of the world will have more individuals with atherosclerotic CVD than any other region [3, 4].

**Fig. 1:**
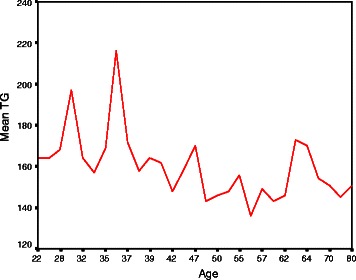
Distribution of Mean Serum Triglycerides (TG) in different age groups

**Fig. 2:**
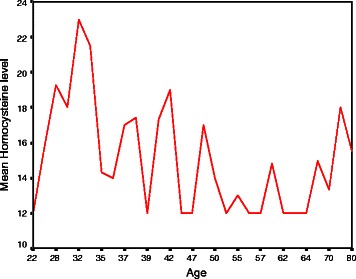
Distribution of Mean Serum Homocysteine in different age groups

South Asian populations have an increased risk and 5–10 years earlier onset for acute myocardial infarction (AMI) compared to Western populations. In recent years, the frequency of AMI in the younger population is increasing [3, 5, 6]. AMI in young individuals can cause death and disability in the prime of life and has serious consequences for the patients, their family and health systems of the nation, causing an increased economic burden. Previous studies have shown that young AMI patients (<40 years) had a high prevalence of smoking, family history and dyslipidemia and a relatively high incidence of normal coronary arteries, non-obstructive stenosis or single-vessel disease [7–9]. Identifying the risk factors for AMI in this group of people is necessary for risk factor modification and developing cost-effective secondary prevention strategies as young AMI patients have different clinical characteristics and pathophysiology from that in older patients [10]. Several studies have documented the classical risk factors for ischemic heart disease (IHD). However, the role of these risk factors in the pathogenesis of IHD and whether they are equally important for the young patients in Bangladesh is still not yet convincingly established. Data on risk factors and in-hospital outcomes for young AMI patients are limited in Bangladesh. We therefore conducted this study to determine the risk factors and in-hospital outcome of AMI among young patients (≤40 years) compared to older (>40 years) patients in Dhaka, Bangladesh. Our hypothesis was that younger patients would have better outcomes and a different pattern of risk factors than older patients.

## Methods

### Study population and setting

We conducted a prospective observational study of 100 patients with AMI attending the Department of Cardiology, National Institute of Cardiovascular Diseases (NICVD), Dhaka between July 2010 and June 2011. We recruited 50 consecutive patients aged less than 40 years and 50 consecutive patients aged 40 years or older. The inclusion criteria were adult patients of both sexes presenting with first acute ST Segment Elevation Myocardial Infarction (STEMI) or AMI within 12 hours onset of chest pain and providing informed consent. The exclusion criteria were: patients with valvular heart disease, congenital heart disease and cardiomyopathy; patients with other major disorders such as severe renal impairment, cancers, systemic infection and those not willing to provide written informed consent. The diagnosis of AMI was established based on the following criteria: detection of rise and/or fall of cardiac biomarkers (preferably troponin) with at least one value above the 99th percentile of the upper reference limit (URL) together with evidence of myocardial ischemia with at least one of the following: symptoms of ischemia; ECG changes indicative of new ischemia (new ST-T changes or new left bundle branch block [LBBB]); development of pathological Q waves in the ECG; imaging evidence of new loss of viable myocardium or new regional wall motion abnormality [11].

### Data collection

All participants presenting at the Emergency Room of NICVD with acute onset of chest pain during the last 12 hours were screened for eligibility by the attending physician. All eligible participants were referred to the study team by the attending physician and first interviewed by a member of the research team at the wards of Cardiology Department, NICVD after the patient’s condition were stable. Blood for biochemical tests were collected from the wards by laboratory assistants experienced in blood collection and sent to NICVD laboratory for analysis. One of the investigator (MAK) performed bedside echocardiography tests in the wards. All participants were followed up during the hospitalization period.

Data were collected using a structured questionnaire and pretested clinical examination form (Supplementary File 1) through face-to-face interviews and clinical examination. The questionnaire contained the following information: demographic data , anthropometric measurements, risk factors (dietary pattern, current tobacco use, family history of premature CAD, history of angina). Dietary pattern was assessed by asking questions on specific food intake. Clinical and laboratory data included: blood pressure (BP), biochemical tests (random blood sugar (RBS), serum creatinine, serum electrolytes, fasting lipid profile, troponin-I, fasting blood sugar (FBS), 2 hours after fasting glucose, fasting plasma total homocysteine level, serum uric acid), electrocardiography (ECG), echocardiography and in-hospital outcomes (as defined below). Weight, height, waist circumference (WC), hip circumference (HC), waist-hip ratio (WHR) was measured using standard clinical guidelines [12]. Body mass index (BMI) was measured as weight in kilograms^2^ divided by height in centimeters. Blood pressure was measured twice (at admission) and the average value was recorded. Echocardiography was done at least two times, first, within 24 hours of admission and last, on the day of discharge, or even more frequently if indicated. Echocardiographic variables included LVID (d), LVID (s), regional wall motion abnormality (RWMA) and left ventricular ejection fraction (LVEF).

### Definition of variables

Hypertension was defined as > 140 mmHg systolic BP or >90 mmHg diastolic BP on at least two occasions or current use of any antihypertensive therapy [13]. Diabetes was diagnosed when patient had classical symptoms of diabetes plus random plasma glucose concentration ≥200 mg/dl (11.1 mmol/L) or FPG ≥126 mg/dl (7 mmol/L) or 2-hr post load glucose ≥ 200 mg/dl (11.1 mmol/L) during an OGTT or using anti-diabetic medications. Dyslipidemia was diagnosed according to ATP-III criteria: LDL cholesterol >100 mg/dl, Total cholesterol > 200 mg/dl, HDL cholesterol <40 mg/dl, triglycerides >150 mg/dl [14]. Family early history of ischemic heart disease (IHD) was considered when any direct blood relative (parents, siblings, children) had any of the following at age <55 years: angina, MI, sudden cardiac death without obvious cause [15]. Cardiogenic shock was defined as evidence of tissue hypo perfusion included by heart failure after correction of preload. Cardiogenic shock was usually characterized by: reduced BP (systolic BP <90 mmHg or a drop of mean arterial pressure >30 mmHg) and/or, low urine output (<0.5 ml/kg/h), pulse rate >60 bpm with or without evidence of organ congestion. For congestive cardiac failure we used Killip classification as follows: Class I: Absence of rales over the lung fields and absence of S3; Class II: Crackles/rales over 50 % or less of the lung fields ± presence of am S3 gallop; Class III: Crackles/rales over >50 % of the lung fields and S3 gallop. Class IV: Cardiogenic shock [15]. BMI was calculated as weight (kg)/height squared (m2) and classified as: underweight < 18.5, normal 18.5 – 24.9, overweight 25.0 – 29.9 and obesity ≥30 [16]. In-hospital outcomes: All patients were followed up during hospitalization and clinical outcome was recorded based on standard criteria as: duration of hospital stay, heart failure, post-MI angina, re-infarction, mechanical complications, significant arrhythmia, cardiogenic shock (as defined below) and death.

### Ethics

Written informed consent was taken from each patient before data collection. Confidentiality was strictly maintained and the patients were informed about the study and their rights to withdraw at any stage which would not hamper the rights to treatments. The study protocol was approved by the institutional review board of National Institute of Cardiovascular Diseases (NICVD), Dhaka.

### Data analysis

Data were analyzed using SPSS version 17 (SPSS Corp. Texas, USA). Data are expressed in frequencies (n), percentage (%), and means ± standard deviation (Mean ± SD). The two study groups were compared using Student’s t-test and Fisher’s exact test for continuous variables and chi-square test for categorical variable, as applicable. Multivariate logistic regression analysis was performed to examine associated risk factors of AMI and in-hospital outcomes controlling for confounding variables (education and socioeconomic status). A *p*-value of less than 0.05 was considered to be statistically significant.

## Results

### Clinical characteristics and biochemical status

Table 1 shows the clinical characteristics and biochemical findings of the study participants. The mean ± SD age of the young and older groups was 36.5 ± 4.6 and 57.0 ± 9.1 years respectively. Majority of patients were male (young 88 % vs. old 68 %). The younger group had a significantly higher proportion of smoking (74 % vs 54 %), family history of IHD (56 % vs 34 %) and higher BMI. In contrast, hypertension (15 % vs 76 %), diabetes (22 % vs 46 %) and history of angina (12 % vs 48 %) were significantly higher among participants of the older group. Mean serum homocysteine and triglyceride were significantly higher in the younger group and mean HDL cholesterol was significantly higher in the older group. The mean C-reactive protein (CRP), uric acid, total cholesterol and LDL cholesterol levels were higher in the younger group, but the difference was not statistically significant. There was no significant difference in mean random blood sugar by age (*p* = 0.37) Figs. 1, 2 .

**Table 1:** Clinical characteristics and biochemical status of study participants (*n* = 100)

Variables	Young group	Older group	*p*-value
	(age ≤ 40) (*n* = 50)	(age > 40) (*n* = 50)	
Clinical characteristics			
Age (Mean ± SD)	36.5 ± 4.6	57.0 ± 9.1	0.001^*^
Male sex	44 (88)	34 (68)	0.02
Smoking	37 (74)	27 (54)	0.04
Chewing tobacco	7 (14)	16 (32)	0.03
Dyslipidemia	26 (52)	20 (40)	0.22
Hypertension	7 (14)	38 (76)	0.001
Diabetes mellitus	11 (22)	23 (46)	0.01
Family history of IHD	28 (56)	17 (34)	0.02
BMI (mean ± SD)	25.21 ± 3.6	24.26 ± 3.61	0.19^a^
Normal (18.5 – 24.9)	23 (56)	28 (56)	0.32
Over weight (25 – 29.9)	16 (32)	14 (28)	0.66
Obese (≥30)	11 (22)	8 (16)	0.44
Waist hip ratio (mean ± SD)	0.96 ± 0.06	0.95 ± 0.05	0.42
Waist hip ratio (>1)	13 (26.0)	9 (18.0)	0.33
History of angina	6 (12)	17 (48)	0.02
Biochemical status			
Serum homocysteine	17.14 ± 5.12	13.84 ± 2.93	0.001
C-reactive protein (CRP)	14.66 ± 6.8	13.02 ± 2.53	0.11
Uric acid	6.7 ± 6.0	5.6 ± 0.6	0.21
Random blood sugar (RBS)	10.6 ± 6.2	11.7 ± 6.0	0.37
Total cholesterol	193.10 ± 21.95	186.58 ± 22.20	0.14
Triglyceride	165.26 ± 23.52	150.40 ± 16.88	0.01
LDL cholesterol	121.69 ± 22.36	113.80 ± 25.05	0.10
HDL cholesterol	38.36 ± 4.11	42.70 ± 4.83	0.01

Values are Mean ± SD or n(%) unless otherwise indicated

^*^p value reached from unpaired t-test

^**a**^chi-square test

### Food habits

The food habits of the study participants are presented in Table 2. The mean frequency of eating rice, beef, chicken and fish was significantly higher in the younger group. The older group reported a significantly higher frequency of eating bread, fruits and vegetables. The younger group reported higher consumption of rice (≥2 times a day), beef ≥2 times per month, chicken ≥2 times per month and fish ≥3 times per month, while the older group reported higher proportion of fruit consumption (≥2 times) and vegetables ≥4 times per week.

**Table 2:** Distribution of study subjects by food consumption

Variables	Young group	Older group	*p*-value
	(age ≤ 40)	(age > 40)	
Rice	50 (100)	50 (100)	
Frequency (per day)	1.98 ± 0.42	1.78 ± 0.54	0.04
1 time	5 (10)	14 (28)	
≥2 times	45 (90)	36 (72)	
Bread	50 (100)	48 (96)	
Frequency (per day)	1.12 ± 0.32	1.28 ± 0.45	0.04
1 time	44 (88)	36 (72)	
2 times	6 (12)	14 (28)	
Beef	25 (50)	9 (18)	
Frequency (per week)	2.04 ± 0.84	1.33 ± 0.50	0.02
1 time	8 (32)	6 (66.7)	
≥2 times	17 (68)	3 (33.3)	
Mutton	4 (8)	2 (4)	
Frequency (per week)	1.50 ± 0.57	2 ± 0.5	0.31
Chicken	43 (86)	47 (94)	
Frequency (per week)	1.33 ± 0.52	1.13 ± 0.33	0.03
1 time	30 (69.8)	41 (87.2)	
≥2 times	13 (30.2)	6 (12.8)	
Fish	50 (100)	50 (100)	
Frequency (per week)	2.80 ± 0.90	1.13 ± 0.80	0.03
1 − 2 times	19 (38)	32 (64)	
≥3 times	31 (62)	18 (36)	
Egg	5 (10)	4 (8)	
Frequency (per week)	2.80 ± 2.3	1.00 ± 1.20	0.18
Milk	4 (8)	3 (6)	
Frequency (per week)	1.25 ± 0.50	1.33 ± 0.57	0.84
Fruits	50 (100)	49 (98)	
Frequency (per week)	1.74 ± 0.66	2.02 ± 0.24	0.02
1 time	19 (38)	1 (2)	
≥2 times	31 (62)	48 (98)	
Vegetables	50 (100)	50 (100)	
Frequency (per week)	3.40 ± 0.83	3.78 ± 0.58	0.01
2 times	9 (18)	4 (8)	
3 times	14 (28)	3 (6)	
>4 times	27 (54)	43 (86)	

values are *n*(%) and mean ± SD

**Table 3:** Echocardiography findings of the study participants (*N* = 100)

Variables	Young group	Older group	*p*-value
	(age ≤ 40) *n* = 50	(age > 40) *n* = 50	
	Number (%)	Number (%)	
Wall involvement			
Anterior MI	23 (46)	22 (44)	NS
Inferior MI	16 (32)	18 (36)	NS
Antero-septal MI	11 (22)	10 (20)	NS
Left ventricular ejection fraction (LVEF)			
LVEF	54.4 ± 7.7	49.8 ± 7.8	0.004
<40	0 (0)	2 (4)	
40 − 49	10 (20)	22 (44)	0.01
≥50	40 (80)	26 (52)	0.003
Incidence of heart failure (Killip classification)			
Class I	2 (4)	5 (10)	0.23
Class II	2 (4)	3 (6)	0.64
Class III	0 (0)	2 (4)	
Class IV	1 (2)	3 (6)	0.30
Pattern of arrhythmia			
CHB	2 (4)	7 (14)	0.08
AF	1 (2)	3 (6)	0.30
VT/VF	2 (4)	3 (6)	0.60
Mechanical complications			
MR	1 (2)	6 (12)	0.05
VSR	0 (0)	1 (2)	

### Echocardiography findings

The frequency of anterior, inferior and anteroseptal MI was similar in both the groups (Table 3). The mean ± SD of left ventricular ejection fraction (LVEF) was significantly higher in the younger group (*p* = 0.004). The incidence of heart failure (according to Killip classification) and presence of any arrhythmia was not significantly different by age. The proportion of mechanical complications of MR was higher in the older group than younger group (p < 0.05).

**Table 4:** Comparison of in-hospital outcome between two groups (*N* = 100)

Variables	Young group	Older group	*p*-value
	(age ≤ 40) (*n* = 50)	(age > 40) (*n* = 50)	
	Number (%)	Number (%)	
Duration of hospital stay (days) (Mean ± SD)	5.08 ± 1.8	10.7 ± 1.8	0.001
Heart failure	5 (10)	13 (26)	0.04
Post MI angina	3 (6)	8 (16)	0.11
Re-infarction	2 (4)	5 (10)	0.23
Significant arrhythmias	5 (10)	13 (26)	0.04
Cardiogenic shock	1 (2)	3 (6)	0.30
Mechanical complications	1 (2)	7 (14)	0.02
Death	1 (2)	6 (12)	0.05

### In-hospital outcomes

Table 4 shows the in-hospital outcome of the study participants. The mean duration of hospitalization was almost double in the older group than in the younger group (*p* = 0.001). The survival rates were higher in younger group but not statistically significant (*p* = 0.05). Older group had significantly worse clinical evolution in terms of higher rates of heart failure, significant arrhythmias and mechanical complications. Fatal outcome due to anterior and inferior MI was less frequent in the younger group (13.64 %) vs. older group (33.33 %) *p* = 0.05.

Table 5 presents the results of logistic regression analysis for risk factors and in-hospital outcomes in young patients. Male sex (OR 3.4, 95 % CI 1.2 to 9.75), smoking (OR 2.4, 95 % CI 1.04 to 5,62), family history of IHD (OR 2.4, 95 % CI 1.11 to 5,54), homocysteine level (OR 1.2, 95 % CI 1.08 to 1.36), taking rice ≥2 times daily (OR 3.5, 95 % CI 1.15 to 10.6) and taking beef (OR 4.5, 95 % CI 1.83 to 11.3) were significant risk factors for development of AMI in the younger group compared to the older group. Older patients had approximately 7.5 times more chance of developing heart failure (OR 7.5, 95 % CI 1.51 to 37.31), 7 times more chance of developing re-infraction (OR 7.0, 95 % CI 1.08 to 45.72), 15 times more chance of developing arrhythmia (OR 15.3, 95 % CI 2.69 to 87.77), 69 times more chance of developing cardiogenic shock (OR 69.0, 95 % CI 5.81 − 85.52) than the younger group, which was statistically significant.

**Table 5:** Logistic regression for risk factors and in-hospital outcomes of AMI in young group

Independent variables	B	Wald	OR	95 % CI		*p* value
				Lower	Upper	
Male sex	1.239	5.454	3.451	1.220	9.759	0.02
Smoking	0.886	4.252	2.425	1.045	5.626	0.04
Family history of IHD	0.904	4.804	2.471	1.110	5.547	0.03
BMI (Overweight)	0.191	0.190	1.210	0.514	2.851	0.66
Homocysteine level	0.198	11.453	1.219	1.087	1.367	0.001
TG	0.036	10.633	1.037	1.014	1.059	0.001
LDL	0014	2.633	1.015	0.997	1.035	0.11
HDL	−0.356	17.316	0.70	0.592	0.826	0.001
Eating rice (≥2 times daily)	1.253	4.883	3.50	1.152	10.633	0.03
Eating beef (per week)	1.516	10.670	4.56	1.834	11.316	0.01
Vegetables **(≥**2 times) weekly	−1.655	11.105	0.191	0.072	0.506	0.001
Heart Failure	2.018	6.102	7.524	1.517	37.311	0.01
Re-infarction	1.952	4.179	7.040	1.084	45.727	0.04
Arrhythmia	2.733	9.464	15.385	2.696	87.776	0.002
Cardiogenic shock	4.234	11.246	69.00	5.809	85.519	0.001

## Discussion

This study, to the best of knowledge, is the first in Bangladesh to demonstrate the risk factors and in-hospital outcomes of AMI in young people. The study shows that majority of young AMI patients were male and a family history of IHD, smoking, overweight, increased homocysteine and triglycerides were the most common risk factors among young patients. Young patients showed a different risk factor profile and better survival rates and in-hospital outcomes compared to the older group.

A majority of our participants were male, which is consistent with previous studies in Bangladesh by which the percentage of male patients were 85 − 92 % [17, 18]. A study by Khan and colleagues with young AMI patients reported smoking (84.4 %), hypertension (46.9 %), dyslipidemia (56.3 %), diabetes (12.5 %), family history (34.4 %) with higher triglyceride level and lower HDL [19]. These findings are consistent with previous studies [8, 19–21]. Another study in Bangladesh showed that AMI in young patients is most commonly seen in males and the most frequent risk factor was smoking [22]. In our study, males had 3.4 times significantly greater chances of developing AMI at younger age compared to females.

Almost half of our participants had higher BMI and one-quarter of young AMI patients had WHR < 1. Younger patients had higher BMI and WHR, but the difference was not statistically significant. Both BMI and WHR are predictors of CVD and mortality [23]. A study in Bangladesh showed an association between hypertension and dyslipidemia [24]. In our study young AMI patients had higher dyslipidemia and lower hypertension than older patients. Our findings support the emphasis on smoking cessation and life-style interventions to prevent CVD among young persons. Previous studies have suggested that in young AMI patients coronary artery spasm might lead to temporary occlusion of the vessel or thrombus or a combination as a result of smoking and dyslipidemia [7, 25]. Therefore, creating awareness for smoking cessation, healthy diet, early screening and interventions such as use of anti-platelet medications and distal protection might be more effective in this group of patients.

Results of our study showed that younger group had higher mean homocysteine, CRP, uric acid and TG and lower HDL cholesterol levels compared to the older group. A study in Bangladesh showed that elevated levels of CRP are significantly and inversely associated with angiographically visible coronary collateral development assessed by Rentrop classification [18]. The study also reported that young patients had significantly higher TG and lower HDL-C, which are known risk factors for AMI [18]. Therefore, our participants might have developed fewer coronary collaterals which might cause premature AMI in this younger group of patients. Previous studies showed significant increase in number of coronary artery involvement by atherosclerotic lesions with increasing levels of plasma homocysteine level [26], which is a strong predictor of mortality in patients with angiographically confirmed coronary artery disease [27]. A study in Bangladesh showed no vessel involvement was more common in young group than older group (21.9 % vs 12.5 %). The younger age group has less favorable lipid profile than older age group having raised total cholesterol, decreased HDL and raised LDL [19].

In this study the mean ejection fraction was significantly higher among young group, as was expected. In our younger and older group, anterior-MI, inferior-MI and antero-septal-MI was 46 % vs. 44 %, 32 % vs. 36 % and 22 % vs. 20 % between groups respectively. A study in Bangladesh among young patients with CVD showed 9.37 % non-Q MI, 28.12 % acute anterior MI, 14.06 % acute anteroseptal-MI, 26.56 % acute inferior-MI, 6.25 % acute infero-posterior-MI [28].

In this study, the younger group reported to consume significantly higher frequency of rice and beef and significantly lower frequency of fruits and vegetables compared to the older group. Previous studies have shown that unhealthy diet rich in carbohydrate and low in fruits and vegetables are a major risk factor for CVD [24, 29, 30]. Dietary results are difficult to compare due to differences in study design and variations of food habits in different countries. However, our result are consistent with other previous studies [31, 32].

AMI in young adults is not as well characterized as in older adults, and limited data suggest that prognosis may be better in this group [33]. Our results showed that AMI was associated with significantly higher mortality and cardiovascular events in the elderly compared with the young, which is similar to an Indian study. [34] In a study by Chowdhury & Marsh, the in-hospital mortality rate among young MI patients was approximately 1 − 6 % compared to 8 − 22 % in the older patients. [35]. Another study showed that AMI in young patients presented with acute onset of symptoms, angiographically complex stenosis morphologic features, and less extensive CAD [36]. AMI in young patients causes significant morbidity, psychological effects, and financial constraints for the person and the family [37]. Screening for risk factors in the young population may help to improve prognosis and prevent AMI in young age [38].

Our result showing better clinical outcome among younger patient is in agreement with previous reports [10, 39]. However, studies in other countries have suggested that although in-hospital outcomes are better in young AMI patients due to less severe coronary vessel involvements, in the long run complications such as history of previous MI, peripheral vascular disease and low ejection fraction are high risks for mortality [40, 41].

### Limitation of the study

Our study has features and limitations that should be kept in mind when using and interpreting its results. First, we conducted an observation study on limited number of patients in a single hospital. Therefore, the results might not be sufficient to change clinical practice or policy recommendations. Further multi-center longitudinal studies with adequate samples and power are recommended. Second, data were collected from one hospital and might not represent the overall AMI population. However, NICVD is the largest tertiary hospital for cardiovascular diseases in the country and patients are referred here for better management from all over Bangladesh. Third, dietary data were collected based on self-reports from patients and recall bias might be a problem. Forth, multiple comparisons were made with limited data and the probability for type I and type II errors can be ruled out as we could not adjust for multiple hypothesis testing. Finally, our patients were not evaluated angiographically and we did not collect the data on percutaneous revascularization and its outcome, which might provide better information.

## Conclusion

This is the first study to present the risk factors and immediate in-hospital outcome of AMI in young patients in Bangladesh. Our results show that young patients with AMI commonly had different risk profile, less extensive MI and better in-hospital survival compared with older patients. Also, young AMI patients had higher prevalence of smoking, family history, unhealthy diet, overweight and dyslipidemia, which are preventable risk factors and should be considered for prevention of AMI in Bangladesh and other developing countries. Further large controlled studies with angiographic exploration and long-term follow up are needed to confirm the pathogenesis of AMI in young patients.
